# RNA Polymerase Activity and Specific RNA Structure Are Required for Efficient HCV Replication in Cultured Cells

**DOI:** 10.1371/journal.ppat.1000885

**Published:** 2010-04-29

**Authors:** Asako Murayama, Leiyun Weng, Tomoko Date, Daisuke Akazawa, Xiao Tian, Tetsuro Suzuki, Takanobu Kato, Yasuhito Tanaka, Masashi Mizokami, Takaji Wakita, Tetsuya Toyoda

**Affiliations:** 1 Department of Virology II, National Institute of Infectious Diseases, Tokyo, Japan; 2 Unit of Viral Genome Regulation, Key Laboratory of Molecular Virology & Immunology, Institute Pasteur of Shanghai, Chinese Academy of Sciences, Shanghai, People's Republic of China; 3 Pharmaceutical Research Lab, Toray Industries, Inc., Kanagawa, Japan; 4 Department of Clinical Molecular Informative Medicine, Nagoya City University Graduate School of Medical Sciences, Aichi, Japan; 5 Research Center for Hepatitis and Immunology, Kohnodai Hospital, International Medical Center of Japan, Chiba, Japan; University of Washington, United States of America

## Abstract

We have previously reported that the NS3 helicase (N3H) and NS5B-to-3′X (N5BX) regions are important for the efficient replication of hepatitis C virus (HCV) strain JFH-1 and viral production in HuH-7 cells. In the current study, we investigated the relationships between HCV genome replication, virus production, and the structure of N5BX. We found that the Q377R, A450S, S455N, R517K, and Y561F mutations in the NS5B region resulted in up-regulation of J6CF NS5B polymerase activity *in vitro*. However, the activation effects of these mutations on viral RNA replication and virus production with JFH-1 N3H appeared to differ. In the presence of the N3H region and 3′ untranslated region (UTR) of JFH-1, A450S, R517K, and Y561F together were sufficient to confer HCV genome replication activity and virus production ability to J6CF in cultured cells. Y561F was also involved in the kissing-loop interaction between SL3.2 in the NS5B region and SL2 in the 3′X region. We next analyzed the 3′ structure of HCV genome RNA. The shorter polyU/UC tracts of JFH-1 resulted in more efficient RNA replication than J6CF. Furthermore, 9458G in the JFH-1 variable region (VR) was responsible for RNA replication activity because of its RNA structures. In conclusion, N3H, high polymerase activity, enhanced kissing-loop interactions, and optimal viral RNA structure in the 3′UTR were required for J6CF replication in cultured cells.

## Author Summary

Hepatitis C virus (HCV) is a major cause of chronic liver disease. Chronic HCV infection affects more than 130 million people worldwide. An efficient cell culture system is indispensable for HCV research and the development of antiviral strategies, including antiviral drugs and vaccines. Using one HCV strain, JFH-1, we have developed a novel cell culture system that, for the first time, has allowed for both the production of infectious HCV and the analysis of the HCV life cycle. To date, JFH-1 is the only HCV strain that replicates efficiently in cultured cells. Understanding the mechanisms underlying replication of JFH-1 in cultured cells is important and advantageous for the development of antiviral strategies. In the present study, we demonstrate that high polymerase activity, enhanced kissing-loop interactions between the NS5B and 3′X regions, and optimal viral RNA structure of the 3′ UTR are required for the efficient replication of JFH-1 and viral production in cultured cells. Our data provides information that will prove essential for the establishment of replication-competent variants of HCV strains that are currently replication incompetent in cultured cells. This study also contributes to a better understanding of the mechanisms underlying persistent HCV infections.

## Introduction

Hepatitis C virus (HCV) contains a positive-stranded RNA genome and belongs to the *Flaviviridae* family [1]. Chronic HCV infection affects more than 130 million people worldwide [2]. The HCV RNA genome is approximately 9.6 kb in length and contains a long open reading frame that encodes a polyprotein of approximately 3,010 amino acids. This polyprotein is processed into at least 10 polypeptides by host and viral proteases [3], [4]. The 5′-untranslated region (UTR) contains a highly conserved internal ribosome entry site (IRES) that is 341 nucleotides long [5]. The 3′UTR is known to contain a variable region (VR), a poly pyrimidine “U/C” (polyU/UC) tract, and a 98-base X-region (3′X tail) [6]. The second stem loop of the X region interacts with the NS5BSL3 cis-acting replication element (CRE) and may contribute to initiation of negative strand RNA synthesis [7].

JFH-1 belongs to genotype 2a and is the only strain that can efficiently replicate and produce virions in HuH-7 and HuH-7-derived cell lines [8], [9], [10]. When the structural protein-coding regions of the non-replicating HCV strains were fused to the non-structural protein-coding region and 3′UTR of JFH-1, replication was initiated and virions were produced in HuH-7-derived cells [10], [11]. In order to analyze the mechanisms underlying the robust replication of JFH-1, we compared JFH-1 with J6CF. J6CF shares approximately 90% sequence homology with JFH-1 but does not replicate in HuH-7 cells. Analysis of JFH-1/J6CF chimeras demonstrated that the NS3 helicase-coding region (N3H) and the NS5B-to-3′X (N5BX) region of JFH-1 conferred replication activity to J6CF in HuH-7 cells [12]. Mutations in the N3H region are expected to affect helicase activity, while mutations in the NS5B-to-3′X region may affect polymerase and replication activity through secondary or higher order structures of the RNA. We have also previously reported that JFH-1-type mutations in the NS5B region enhanced genotype 1b RdRP activity *in vitro* [13]. Thus, JFH-1-type mutations in the NS5B region of J6CF are hypothesized to enhance J6CF RdRP activity. As mentioned above, the 3′UTR of the HCV genome consists of a VR, polyU/UC tracts of various lengths and a highly conserved 3′X tail. Deletion of the VR was reported to allow replication in both cultured cells [14] and in the chimpanzee [15]. The minimum length of polyU/UC tract required for replication has also been previously determined [14], [16].

In the current study, we examined RNA polymerase activity and the RNA structures of the NS5B and 3′UTR that contribute to HCV replication, and determined the essential domains required for robust HCV RNA replication in cultured cells.

## Materials and Methods

### Cell culture

HuH-7 cells [17] and Huh-7.5.1 cells [9] were cultured at 37°C in Dulbecco's modified Eagle's medium containing 10% fetal bovine serum under 5% CO_2_ conditions.

### Construction of plasmids encoding a C-terminal 12xHis tagged HCV RdRP lacking 21 C-terminal amino acids

HCV JFH-1 and J6CF RdRP without the C-terminal 21 amino acid hydrophobic sequence were PCR amplified from pJFH1 [8] and pJ6CF (a kind gift from Jens Bukh) [15], respectively. Primer sequences for mutagenesis are listed in Table S1. Following digestion with X*ba*I and X*ho*I, DNA fragments were cloned into the N*he*I and X*ho*I sites of pET21b (Novagen, Madison, WI), resulting in pET21bHCVJFH-1RdRpwt and pET21bHCVJ6CFRdRpwt. pET21bHCVJFH-1RdRpwt and pET21bHCVJ6CFRdRpwt were then digested with X*ba*I and X*ho*I and the RdRP fragments cloned into the same restriction sites of pET28a, resulting in pET21(KM)JFH-1RdRpwt and pET21(KM)J6CFRdRpwt, respectively.

### Mutation analysis of J6CF and JFH-1 RdRP

JFH-1-type substitutions (S377R, A450S, S455N, R517K, and Y561F in the NS5B region; amino acid numbers are based on the AA relative numbering [18]) were introduced into J6CF RdRP and J6CF-like substitutions (S450A, N455S, K517R, F561Y, and F561I) and D318A were introduced into JFH-1 RdRP using the QuickChange II Site-Directed Mutagenesis Kit (Stratagene, La Jolla, CA). Primer sequences for mutagenesis are listed in Table S1. Sequences were confirmed by nucleotide sequencing.

### Expression, purification, and *in vitro* transcription of HCV RdRP

pET21(KM)JFH-1RdRPwt, pET21(KM)J6CFRdRPwt, and their mutants were expressed with pGEX-HSP90α [13] in *Escherichia coli* Rosetta/pLysS (Novagen). RdRP was then purified as previously described [13], with the exception that protein induction was undertaken at 18°C for 4 h. *In vitro de novo* transcription was performed as described previously [13]. Briefly, following 30 min pre-incubation without ATP, CTP, or UTP, 0.1 µM HCV RdRP was incubated in 50 mM Tris/HCl (pH 8.0), 200 mM monopotassium glutamate, 3.5 mM MnCl_2_, 1 mM DTT, 0.5 mM GTP, 50 µM ATP, 50 µM CTP, 5 µM [α-^32^P]UTP, 0.02 µM RNA template (SL12-1S) and 100 U/ml human placental RNase inhibitor at 29°C for 90 min. [^32^P]-RNA products were subjected to PAGE (6% gel, 8 M urea). The resulting autoradiograph was analyzed with a Typhoon trio plus image analyzer (GE Healthcare, Piscataway, NJ). The radio isotope count of 184 nt RNA product of each mutant RdRPs was measured and compared to that of JFH-1 RdRP wt in the same PAGE.

### Subgenomic-replicon constructs

pSGR-J6/N3H+5BSLX-JFH1/Luc was constructed by replacement of the 5BSL-to-3′X fragment (9211 to 9678 of JFH-1) generated by PCR with the corresponding fragment of pSGR-J6/N3H+3′UTR-JFH1/Luc [12]. Constructs with substitutions in NS5B region were generated as follows; mutations were introduced by PCR-based mutagenesis and *Xho*I-*Xba*I-restricted fragments were exchanged with the corresponding fragment of pSGR-J6/N3H+5BSLX-JFH1/Luc or pSGR-J6/N3H+3′UTR-JFH1/Luc [12]. To generate the constructs used for the analyses of the 3′UTR, VR fragments (9415–9479 of JFH-1 and J6CF) or polyU/UC fragments (9480–9579 of JFH-1 and 9480–9606 of J6CF) were generated by PCR and replaced with the corresponding fragment of pSGR-J6/N3H+5BSLX-JFH1/Luc. To generate the constructs with substitutions in the VR or 3′SL2, mutations were introduced by PCR-based mutagenesis and *Sgr*AI-*Xba*I-restricted fragments were exchanged with the corresponding fragment of pSGR-J6/N3H+5BSLX-JFH1/Luc. Primer sequences for mutagenesis are listed in Table S1.

### Full-length genomic HCV constructs

Plasmids used in the analysis of genomic RNA replication and core production were constructed from pJ6/N3H+N5BX-JFH1 [12] and pJ6CF [15]. pJ6/N3H+5BSLX-JFH1 was constructed by replacement of the corresponding sequence with the 5BSL-to-3′X fragment (9211 to 9678 of JFH-1) generated by PCR. pJ6/N3H+3′UTR-JFH1 was constructed by using the N3H region [*Cla*I (3929) - *Eco*T22I (5293)] and 3′UTR [*Stu*I (9415) - *Xba*I (9678)] of JFH-1 to replace the corresponding sequences of pJ6CF. Mutagenesis was performed as described above.

### RNA synthesis and transfection

RNA synthesis and transfection were performed as described previously [8], [12]. Briefly, plasmids were linearized with *Xba*I, treated with Mung Bean Nuclease (New England Biolabs, Ipswich, MA) and purified. Linearized, purified DNA was then used as a template for *in vitro* RNA synthesis using the MEGAscript T7 kit (Ambion, Austin, TX) in accordance with the manufacturer's instructions. Synthesized RNA was treated with DNase I (Ambion) followed by purification using ISOGEN-LS (Nippon Gene, Tokyo, Japan). The quality of the synthesized RNA was examined via agarose gel electrophoresis. Ten micrograms of *in vitro*-synthesized RNA was used for each electroporation. Trypsinized HuH-7 cells or Huh-7.5.1 cells (3×10^6^ cells) were washed with Opti-MEM I (Invitrogen, Carlsbad, CA) and resuspended in Cytomix buffer [19]. RNA was then combined with 400 µl of cell suspension and the mixture was transferred to an electroporation cuvette (Bio-Rad, Hercules, CA). The cells were then pulsed at 260 V and 950 µF using the Gene Pulser II apparatus (Bio-Rad). Transfected cells were immediately transferred to 6-well plates containing culture medium and incubated at 37°C under standard 5% CO_2_ conditions.

### Luciferase reporter assay

Luciferase activity of the JFH-1 subgenomic replicon and chimeras in HuH-7 cells were measured as described previously [12], [20]. Briefly, 5 µg of transcribed RNA was transfected into 3×10^6^ HuH-7 cells by electroporation. Transfected cells were immediately resuspended in culture medium and seeded into 6-well culture plates. Cells were then harvested at 4, 24, and 48 h after transfection and lysed with 200 µl of Cell Culture Lysis Reagent (Promega, Madison, WI). Debris was removed by centrifugation. Luciferase activity was quantified using a Lumat LB9507 luminometer (EG & G Berthold, Bad Wildbad, Germany) and a Luciferase Assay System (Promega). Assays were performed three times independently, with each value corrected for transfection efficiency as determined by measuring luciferase activity 4 h after transfection. Data are presented as relative light units (RLU).

### Quantification of HCV core protein

To estimate the concentration of HCV core protein in the culture medium, we harvested supernatants at the indicated time points. The supernatant was then passed through a filter with a 0.22- µm pore size (Millipore, Bedford, MA) and subjected to the chemiluminescence enzyme immunoassay (Lumipulse II HCV core assay, Fujirebio, Tokyo, Japan) in accordance with the manufacturer's instructions.

### Infection of cells with secreted HCV and determination of infectivity

Culture medium from RNA transfected cells was collected at 72 hours post-transfection. Huh7.5.1 cells were seeded at a density of 1x10^4^ cells per well in poly-*D*-lysine coated 96-well plates (CORNING, Corning, NY). On the following day, the collected culture media were serially diluted and used for inoculation of the seeded cells, and the plates were incubated for another 3 days at 37°C. The cells were fixed in methanol for 15 min at −20°C, and the infected foci were visualized by immunofluorescence as described below.

Cells were blocked for 1 hour with BlockAce (Dainippon Sumitomo Pharma, Osaka, Japan), then washed with PBS, followed by incubation with anti-core antibody at 50 µg/ml in BlockAce. After incubation for 1 hour at room temperature, the cells were washed and incubated with a 1∶400 dilution of AlexaFluor 488-conjugated anti-mouse IgG (Molecular Probes, Eugene, OR) in BlockAce. The cells were then washed and examined using fluorescence microscopy (Olympus, Tokyo, Japan). Infectivity was quantified by counting the infected foci and expressed as focus forming units per milliliter (ffu/ml).

### Chemicals and radio isotope

Nucleotides were purchased from GE, [**α**-^32^P]UTP from New England Nuclear (Boston, MA), and human placental RNase inhibitor and restriction enzymes from TaKaRa (Shiga, Japan).

### Statistical analysis

Significant differences were evaluated using the Student's *t*-test. p<0.05 was considered significant.

### RNA secondary structure prediction

RNA secondary structure prediction was performed using Mfold software [21].

## Results

As we have reported previously, the NS3 helicase and the NS5B-to-3′X regions of JFH-1 are important to confer replication competence to J6CF, a replication-incompetent strain [12]. Of these two regions, NS5B-to-3′X of JFH-1 is the most important to replication-competence. The NS5B region encodes RdRP, and the JFH-1-version of this polymerase may have high activity and be crucial to replication-competence. The requirement of 3′UTR of JFH-1 suggested that the RNA structure in this region is important for efficient genome replication. To understand the mechanisms of efficient replication of JFH-1 in HuH-7 cells, we focused on the NS5B-to-3′X region because the NS3 helicase region of JFH-1 had relatively minor effects on replication of J6CF derivatives [12]. In order to identify the important protein domains within RdRP required for efficient virus replication, we compared the RNA polymerase activity of HCV J6CF RdRP to that of JFH-1 RdRP using three assays, *in vitro* transcription with purified RdRP, *in vivo* virus RNA replication, and *in vivo* virus production. To identify the important sequences or structures in the NS5B-to-3′X region involved in efficient replication, we analyzed the effect of sequence differences in this region on replication of the viral genome.

### Comparison of RNA polymerase activity *in vitro*

By comparing the sequence of RdRP of JFH-1 (GenBank Accession No. AB047639), J6CF (AF177036), other 2a strains (AB047640 – 5, AY746460, AF238481 - 5, AF169002 -5), a 1a strain (H77: AF009606), and four 1b strains (Con1: AJ238799, AB080299, AY045702, M58335), we found 14 amino acids variants unique to JFH-1 RdRP (57T, 130P, 131Q, 150A, 377R, 405I, 435V, 450S, 455N, 474M, 479H, 517K, 561F and 571S). We focused on five JFH-1-type amino acid substitutions (Q377R, A450S, S455N, R517K, and Y561F) that have been shown to increase the polymerase activity of 1b RdRP [13]. We introduced these JFH-1-type amino acid substitutions into J6CF RdRP, individually and in combination, to test their effects on polymerase activity. We also tested a J6CF RdRP variant with a R517K substitution because it was included in the J6/N3H+5BSLX-JFH1 replicon (see below), although it did not enhance the polymerase activity of 1b RdRP *in vitro* [13].

**Figure 1:**
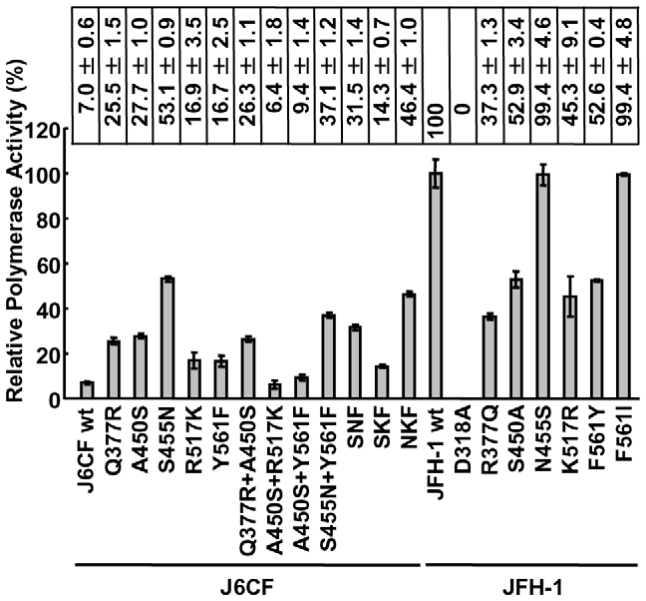
Relative HCV RNA polymerase activity of JFH-1 and J6CF wild-type and mutant RdRP. HCV RdRP activity was measured using the purified HCV RdRP (Fig. S1A) and the average RdRP activity and the standard deviation (error bar) relative to that of JFH-1 RdRP wt were calculated from three independent experiments (Representative gel images are shown in Fig. S1B). The relative activity values are presented above the graph. SNF, A450S+S455N+Y561F; SKF, A450S+R517K+Y561F; NKF, S455N+R517K+Y561F.

The RdRPs of HCV JFH-1 and J6CF and mutant variants were purified as indicated in the Materials and Methods and Fig. S1A. The polymerase activity of wild-type (wt) and mutant RdRPs was measured using a *de novo* transcription system (Fig. 1 and Fig. S1B). The activity of J6CF RdRP was 7.0±0.6% of that of JFH-1. Similar to results seen with 1b RdRP substitution variants, the single amino acid substitutions Q377R, A450S, S455N, R517K, and Y561F resulted in increased polymerase activity of J6CF RdRP (25.5±1.5, 27.7±1.0, 53.1±0.9, 16.9±3.5 and 16.7±2.5% of JFH-1 RdRP wt, respectively). However, combining double and triple amino acid substitutions did not demonstrate any additive or synergistic effects on the *in vitro* polymerase activity (Fig. 1).

JFH-1 RdRP variants with individual J6CF-type amino acid substitutions, including R377Q, S450A, N455S, K517R, and F561Y, were also examined *in vitro*. With the exception of N455S, all other J6CF-type amino acid substitutions reduced the activity of JFH-1 RdRP, with levels ranging from 37.3 to 52.9% of the activity from wt JFH-1 RdRP (Fig. 1). The N455S variant maintained polymerase activity similar to that of JFH-1 wt. The JFH-1 D318A variant has a mutation in the active site of RdRP and showed no polymerase activity, confirming our *in vitro* transcription system.

### JFH-1-type amino acid residues in the NS5B region restored the replication activity of the J6CF-based replicon

**Figure 2:**
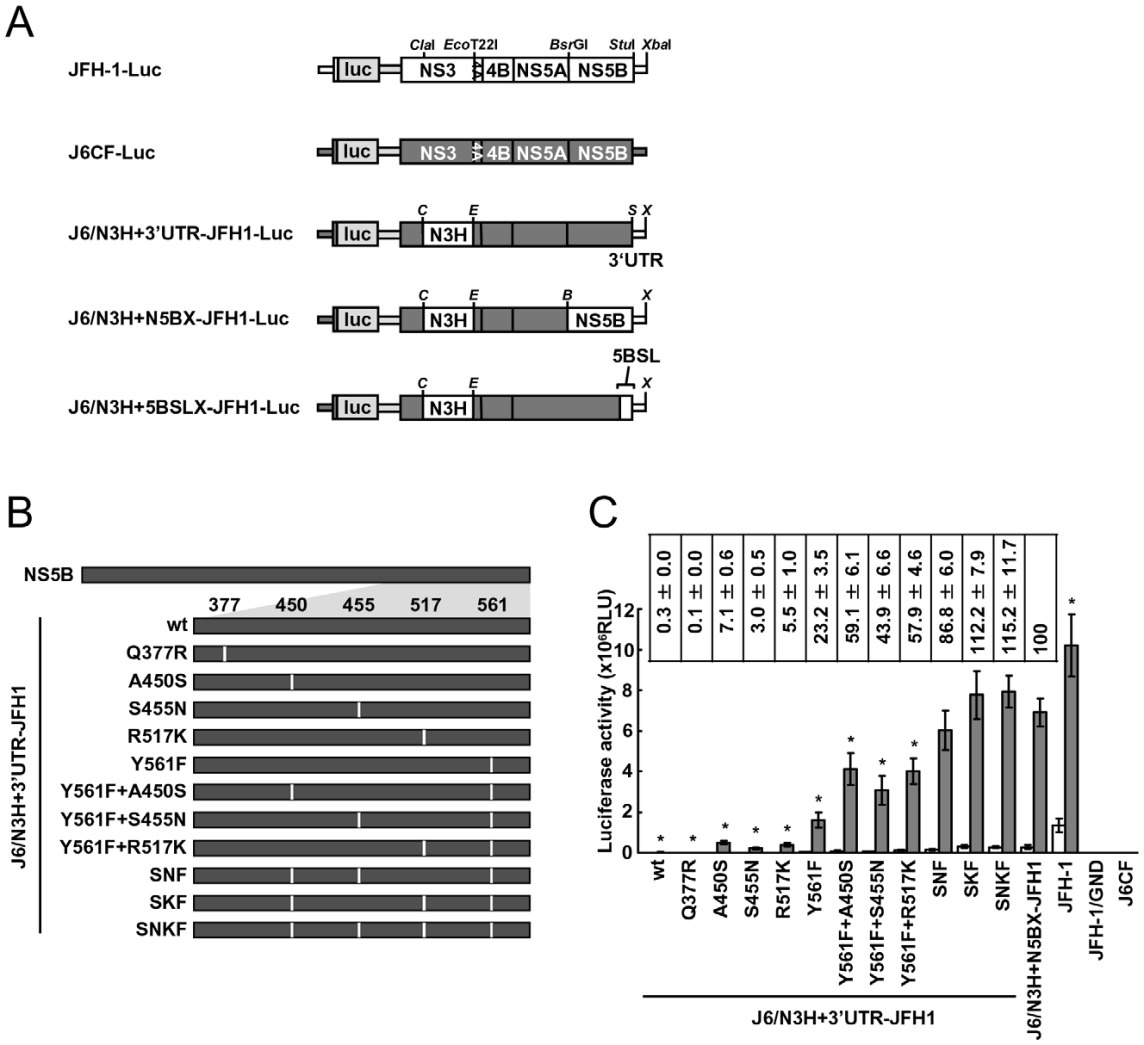
Luciferase activity of J6CF backbone replicons containing substitutions in the NS5B region. (A) Schematic structures of wt JFH-1 and J6CF constructs and the chimeric subgenomic replicons containing a J6CF backbone. The restriction enzyme recognition sites used for the construction of plasmids are indicated. *C*, *Cla*I; *E*, *Eco*T22I; *B*, *Bsr*GI; *S*, *Stu*I; *X*, *Xba*I; wt, wild-type. (B) Schematic diagram of the mutations introduced into J6/N3H+3′UTR-JFH1-Luc and J6/N3H+3′UTR-JFH1. (C) Replication activity of J6CF-based replicons. Subgenomic RNA was synthesized *in vitro* from wild-type or chimeric replicon constructs. Transcribed subgenomic RNA (5 µg) was then electroporated into HuH-7 cells and the cells harvested at 4, 24, and 48 h after transfection. The harvested cells were lysed, and the luciferase activity in the cell lysates was measured. The assays were performed three times independently and the results expressed as luciferase activities (RLU). Each value was corrected for transfection efficiency as determined by measuring the luciferase activity 4 h after transfection. Data are presented as the mean ± standard deviation for luciferase activity at 24 h (white bars) and 48 h (gray bars) after transfection. Asterisks indicate significant differences relative to the replication activity of J6/N3H+N5BX-JFH1 (p<0.05) at 48 h and the values represent the relative values against J6/N3H+N5BX-JFH1 at 48 h after transfection. SNF, A450S+S455N+Y561F; SKF, A450S+R517K+Y561F; SNKF, A450S+ S455N+R517K+Y561F.

In order to test whether the JFH-1-type amino acids substitutions into the NS5B region of J6CF that enhanced polymerase activity *in vitro* enabled the replication of J6CF in cultured cells, we used the subgenomic J6CF replicon harboring the NS3 helicase region and 3′UTR of JFH-1 (J6/N3H+3′UTR-JFH1-Luc; Fig. 2A) as a reference construct. This replicon could replicate in cultured cells but exhibited less than 1% of the JFH-1 replication activity [12]. In order to test the effect of JFH-1 type amino acids on replication, we introduced the five substitutions that increased polymerase activity of J6CF RdRP *in vitro* (Q377R, A450S, S455N, R517K, and Y561F, see Fig. 2B) into the subgenomic replicon J6/N3H+3′UTR-JFH1-Luc and analyzed their effects on RNA replication. Among these JFH-1-type amino acids substitutions, Y561F was the most effective (23.2±3.5% of J6/N3H+N5BX-JFH1-Luc; Fig. 2C), while A450S, S455N, and R517K exhibited only a small effect on the replication activity (7.1±0.6%, 3.0±0.5%, and 5.5±1.0% of J6/N3H+N5BX-JFH1-Luc, respectively; Fig. 2C). The Q377R mutation demonstrated no effect on replication (Fig. 2C). We next tested the effects of Y561F in combination with each of the other substitutions. We found that A450S, S455N, and R517K mutations enhanced the replication activity of Y561F (59.1±6.1%, 43.9±6.6%, and 57.9±4.6% of J6/N3H+N5BX-JFH1-Luc, respectively; Fig. 2C). We also tested the effects of triple mutations and found that the replication activity of the SNF (A450S+S455N+Y561F) and SKF (A450S+R517K+Y561F) mutants demonstrated 86.8±6.0% and 112.2±7.9% replication activity of J6/N3H+N5BX-JFH1-Luc, respectively (Fig. 2C). In addition, we did not observe any significant differences between replicon activity of these mutants and that of J6/N3H+N5BX-JFH1-Luc. A combination of four mutations (SNKF; A450S+S455N+R517K+Y561F) resulted in similar activity as SKF (115.2±11.7% of J6/N3H+N5BX-JFH1-Luc; Fig. 2C). These results indicated that Y561F represented the most effective JFH-1-type mutation required for efficient replication, and that SKF and SNKF were sufficient to support replication activity equivalent to that of the replicon with the entire NS5B and 3′ UTR of JFH-1 (J6/N3H+N5BX-JFH1-Luc). The additive effects of the JFH-1-type NS5B substitutions on the replicon differed from results obtained with the *in vitro* polymerase activity assay.

**Figure 3:**
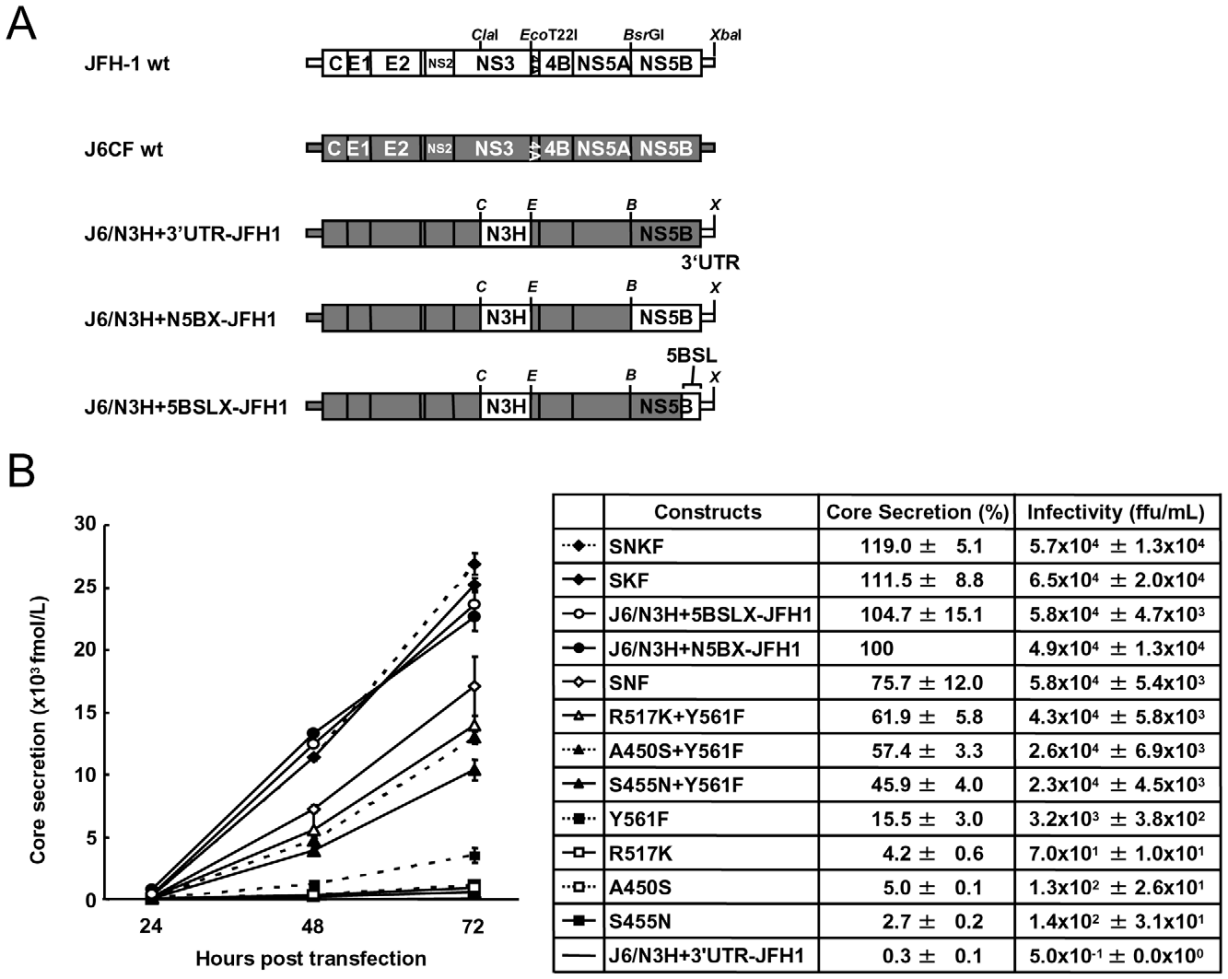
Analysis of transient replication of genomic chimeric HCV RNA. (A) The structure of full-length chimeric HCV RNA. Each chimeric full-length construct was prepared by the replacement of the indicated restricted fragments. The restriction enzyme recognition sites used for the plasmid constructions are indicated. *C*, *Cla*I; *E*, *Eco*T22I; *B*, *Bsr*GI; *S*, *Stu*I; *X*, *Xba*I; wt, wild-type. (B) HCV core protein production in the culture medium from RNA-transfected cells. Transcribed wild-type or chimeric full-length HCV RNA (10 µg) was transfected into Huh-7.5.1 cells. Culture medium was harvested at 4, 24, 48, and 72 h after transfection. The amount of core protein in the harvested culture medium was measured using a HCV core chemiluminescence enzyme immunoassay (Lumipulse II HCV core assay). The assays were performed three times independently, and the data are presented as the mean ± standard deviation. Values in the right panel represent the relative core values against J6/N3H+N5BX-JFH1 at 72 h after transfection and infectious titers of the media from chimeric HCV RNA-transfected cells at 72h after transfection determined using Huh7.5.1 cells. SNF, A450S+S455N+Y561F; SKF, A450S+R517K+Y561F; SNKF, A450S+ S455N+R517K+Y561F.

Next, we examined whether these substitutions were sufficient for full-genome RNA replication and virus production. We used Huh-7.5.1 cells to assess virus production because Huh-7.5.1 is highly permissive for HCV propagation [9]. We found that J6/N3H+3′UTR-JFH1-Luc showed weak replication activity (Fig. 2C), and the core protein was not detectable in the culture medium of J6/N3H+3′UTR-JFH1-transfected cells (Fig. 3B). The constructs expressing A450S, S455N, or R517K substitution variants demonstrated only very low core levels in the supernatant, while the construct expressing the Y561F mutation underwent RNA replication and produced the core protein (Y561F; 15.5±3.0% of J6/N3H+N5BX-JFH1; Fig. 3B). Double mutants containing the Y561F mutation were found to produce greater amounts of core protein than the Y561F single mutant (A450S+Y561F, 57.4±3.3%; S455N+Y561F, 45.9±4.0%; and R517K+Y561F, 61.9±5.8% of J6/N3H+N5BX-JFH1; Fig. 3B). The triple mutant SNF (A450S+S455N+Y561F) produced more core protein than the double mutants (75.7±12.0% of J6/N3H+N5BX-JFH1; Fig. 3B). In addition, we observed that the core production from the SKF and SNKF mutant RNA-transfected cells was similar to the levels produced by J6/N3H+N5BX-JFH1 (111.5±8.8% and 119.0±5.1% of J6/N3H+N5BX-JFH1, respectively; Fig. 3B). We also measured infectivity of the supernatants from the mutant RNA-transfected cells at 72h after transfection (Fig. 3B). The levels of infectious titers correlated with the core levels among the tested constructs in this experiment. These results indicated that the SKF substitutions in the C-terminal region of NS5B were sufficient to elevate viral RNA replication and viral production.

### Extra complementary sequence at the 5BSL3.2 kissing-loop interaction site of JFH-1 was essential for efficient replication

**Figure 4:**
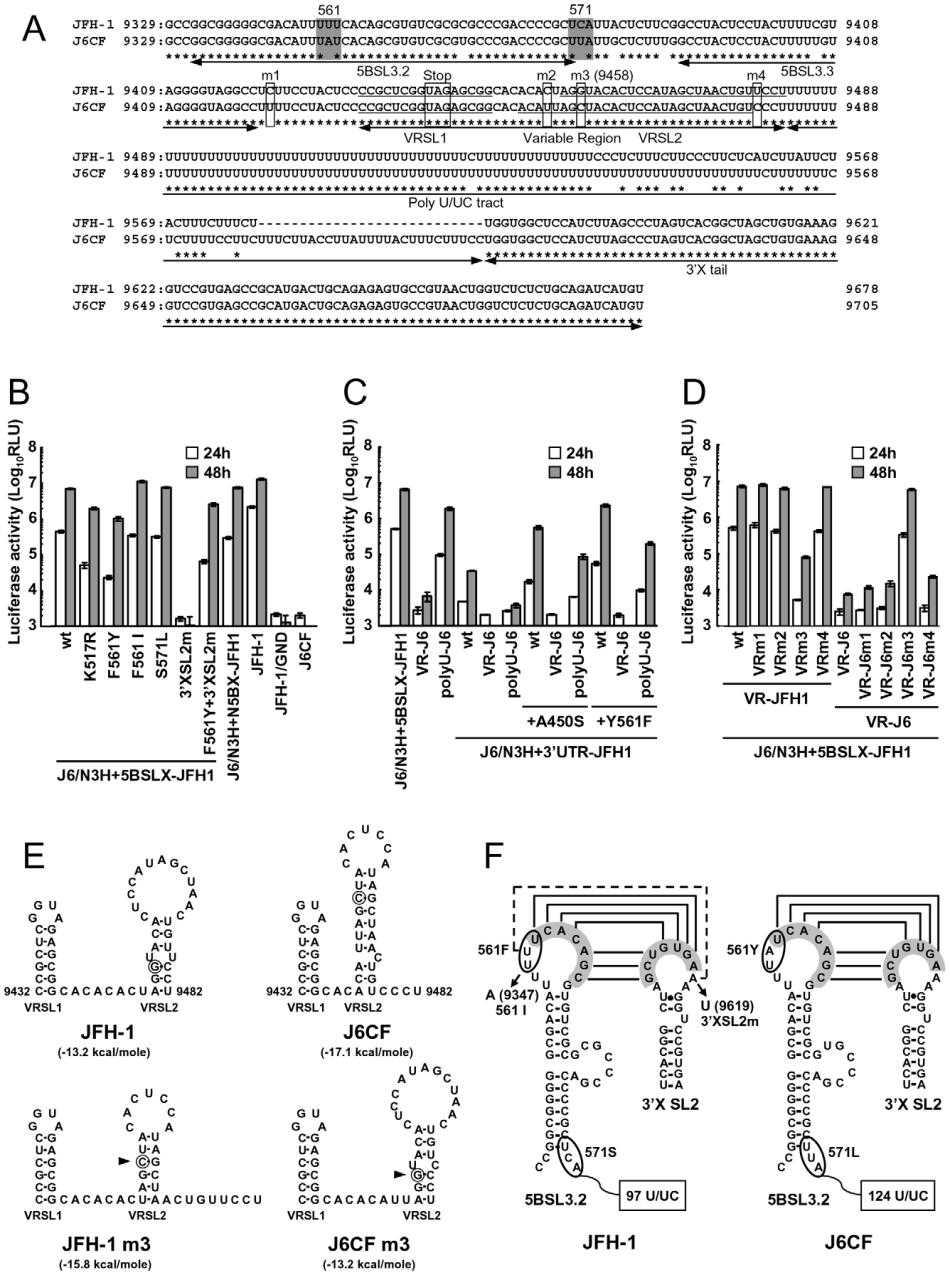
Replication activity of J6CF-based replicons containing variants or substitutions. (A) Comparison of the nucleotide sequence of 5BSL3.2 to 3′X of JFH-1 and J6CF. Boxes indicate nucleotide differences in VR and stop codon. Shaded boxes indicate non-synonymous variants in this region. 5BSL3.2, 5BSL3.3, Variable Region (VR), Poly U/UC tract, and 3′X tail are indicated by double-headed arrows in the figure. Stem-loop structures of VR (VRSL1 and VRSL2) are underlined. Asterisk; conserved nucleotides between JFH-1 and J6CF. (B, C, D) Replication activity of J6CF-based replicons. Five micrograms of *in vitro* synthesized RNA was electroporated into HuH-7 cells and the cells were harvested at 4, 24, and 48 h after transfection. The harvested cells were then lysed, and the luciferase activity in the cell lysates was measured. The assays were performed three times independently, and the results were expressed as luciferase activities (RLU). Data are presented as the mean ± standard deviation for luciferase activity at 24 h (white bars) and 48 h (gray bars) after transfection. (E) The predicted secondary structure of the VR. The RNA secondary structures of JFH-1, JFH-1 m3, J6CF, and J6CF m3 were predicated by Mfold. The stem-loop structure 1 (VRSL1) and 2 (VRSL2) are indicated. Nucleotide 9458 is circled and the mutated nucleotides are indicated by arrowheads. (F) Schematic structures of the 5BSL3.2 and X tail. The predicted stem loop structure of 5BSL3.2 and SL2 of 3′X of JFH-1 and J6CF strains are indicated. The sequences forming kissing interaction with 3′X SL2 [22] are shaded. Codons encoding aa 561 and 571 are circled and the mutated sequences are indicated. The reported kissing-loop interactions are indicated by the connecting lines. The predicted interaction of the JFH-1 strain is indicated by the dotted connecting line.

We observed a discrepancy between the *in vitro* RNA polymerase activity assay and the genome replication assay in the effects of the amino acid substitutions (Figs. 1 and 2C). Y561F was the most effective JFH-1-type amino acid substitution in the replication assay, while S455N was the most effective in the *in vitro* polymerase activity assay. As the kissing-loop interaction between 5BSL3.2 and 3′X are important for RNA replication and amino acid (aa) 561 encoding nucleotides are involved in the stem-loop 3.2 in the NS5B region (5BSL3.2) [7], [16], [22], we hypothesized that the cis-factor (genome structure) may also affect RNA replication in the cells. Thus, we constructed the subgenomic replicon J6/N3H+5BSLX-JFH1-Luc and the full genome construct J6/N3H+5BSLX-JFH1 that contained the NS3 helicase region and the 5BSL3-to-3′X region (nucleotide (nt) 9211 to 9678) of JFH-1 (Figs. 2A and 3A), and determined their replication activity and virus production level. As presented in Figure 4B, the J6/N3H+5BSLX-JFH1-Luc construct demonstrated similar replication activity to that of J6/N3H+N5BX-JFH1-Luc 48h post-transfection (92.9±7.5% of J6/N3H+N5BX-JFH1; Fig. 4B). Moreover, both J6/N3H+N5BX-JFH1 and J6/N3H+5BSLX-JFH1 released similar levels of core protein into the supernatant (Fig. 3B).

We next analyzed the effects of mutations in the J6/N3H+5BSLX-JFH1 construct. The 5BSL region of this construct contains three amino acid differences from J6CF (R517K, Y561F, and L571S). R517K and Y561F were important in the *in vitro* polymerase activity assay (Fig. 1). We did not assess aa 571 *in vitro* because it was deleted to purify HCV RdRP. The replication activities of J6/N3H+5BSLX-JFH1-Luc with K517R or F561Y were found to be 28±2.7% and 14±2.0% of J6/N3H+5BSLX-JFH1-Luc, respectively, confirming the importance of these JFH-1-type amino acids for replication (Fig. 4B). J6/N3H+5BSLX-JFH1-Luc with S571L revealed similar replicon activity as the J6/N3H+5BSLX-JFH1-Luc (108±7.8% of J6/N3H+5BSLX-JFH1 wt; Fig. 4B). These results indicated the importance of 517K and 561F but not 571F in the 5BSL region of JFH-1 in efficient RNA replication. The codon encoding aa 561 possibly affects RNA structure, as it is located in the loop of stem-loop 3.2 in NS5B (5BSL3.2) and overlaps sequences important to the kissing-loop interaction with the stem loop 2 of the 3′X region (3′X SL2) [22]. Although we demonstrated that aa 561F was more effective than 561Y in RdRP activity *in vitro* (Fig. 1), it remains possible that the nucleotide mutation located at the codon of aa 561 affected the RNA structure and genome replication, as the replication activity of J6/N3H+3′UTR-JFH1-Luc with Y561F was the highest of all the clones with JFH-1 type single amino acids in the NS5B region (Fig. 2C). To investigate the effects of these mutations on RNA structure, we made mutants with nucleotide substitutions at the codon of aa 561 (Fig. 4F). The codon encoding aa 561 was UUU (Phe) for JFH-1 and UAU (Tyr) for J6CF. The third base of the codon overlaps with the kissing sequence [22]. In order to maintain the 5BSL3.2 stem loop structure and the kissing interaction between 5BSL3.2 and 3′X SL2, the third base should be U (nt 9349 of JFH-1). JFH-1 may exhibit additional interactions between 9348U of 5BSL3.2 and 9619A of 3′X SL2 to enhance kissing-loop interaction. To assess this hypothesis, we fixed the second base (9348) as U, and the first base (9347) was altered from U to A, G or C. The G and C substitutions were predicted to disrupt the important loop structure of 5BSL3.2 using Mfold and considered to affect replication activity. We next investigated the effects of U to A substitution (AUU, F561I) in an *in vitro* assay. F561I was introduced into JFH-1 RdRP and its RdRP activity was 99.4±4.8% of the wt, demonstrating that an F to I mutation did not affect polymerase activity (Fig. 1). We also examined the effects of the F561I mutation on RNA replication in the cells, and it revealed that it had similar replication activity as the wt, confirming that this mutation exhibited no effect on RNA replication in cultured cells (Fig. 4B). These results demonstrated that both Phe and Ile could be substituted at aa 561 and revealed the importance of the precise RNA structure of this region. Finally, we introduced an A to U mutation at nt 9619 in the 3′X SL2 that was complementary to the second base of the codon encoding 561F (9348) to alter the kissing-loop interaction (Fig. 4F; 3′XSL2m). We observed a significant reduction in 3′XSL2m replication activity (Fig. 4B; 3′XSL2m). However, when 3′XSL2m was combined with the F561Y mutation that was expected to recover the kissing-loop interaction, replicon activity was restored (Fig. 4B; F561Y+3′XSL2m). These results indicated that the extra complementary sequence at the kissing-loop interaction site of 5BSL3.2 was important for the efficient RNA replication of JFH-1. The extra complementary sequence may enhance the kissing loop interactions. We also tested the effect of the Y561F substitution on replicons of other genotypes, H77S (GT1a) and HCV-N (GT1b). While the Y561F substitution increased replication activity in both genotype 1 strains (Text S1 and Fig. S3), the Y561F effect on the genotype 1 strains was much smaller than its corresponding effect on J6CF.

### A shorter poly U/UC sequence in the JFH-1 strain favored replication

We next compared the sequences of the poly U/UC tracts of the 3′UTRs of JFH-1 and J6CF. The poly U/UC tract of JFH-1 was 27 nucleotides shorter than that of J6CF (Figs. 4A and F). The polyU stretch of the pJ6CF plasmid that we used was six nucleotides shorter than that of the original J6CF sequence reported ([15], GenBank: AF177036). In order to analyze the effects of poly U/UC length on HCV replication, the poly U/UC region of J6/N3H+5BSLX-JFH1-Luc was replaced with that of J6CF and was designated as polyU-J6. The replicon activity of J6/N3H+5BSLX-JFH1-Luc with polyU-J6 was approximately four times lower than that of the J6/N3H+5BSLX-JFH1-Luc (Fig. 4C). This result showed that longer polyU/UC region lengths of J6CF were not favorable for efficient replication.

### JFH-1 type structure of the variable region was advantageous for efficient replication

When we compared the VR sequences of the 3′UTRs of JFH-1 and J6CF, we found that four nucleotides are different between the VRs of JFH-1 and J6CF and that substitution of the VR from JFH-1 with that of J6CF of J6/N3H+5BSLX-JFH1 resulted in a 1000-fold decrease in replication activity (Fig. 4C, VR-J6). Mfold analysis of predicted RNA secondary structure of the VR in JFH-1 and J6CF suggests that there are two stem-loop structures in the VR. The first stem loop (VRSL1) structure is identical in JFH-1 and J6CF, but the loop of the second stem-loop (VRSL2) is larger in JFH-1 than in J6CF (Fig. 4E). Analysis of the effects of these nucleotide mutations on RNA structure revealed that only the third mutation (m3 at 9458 in Fig. 4A) is predicted to alter the structure of VRSL2 (Fig. 4E). The m3 G substitution into J6CF VR generated a predicted structure identical to that of JFH-1 VRSL2 resulting in identical VR structures (Fig. 4E). The m3 C substitution altered the structure of JFH-1 VR to the J6CF type (Fig. 4E). Substitutions of other nucleotides did not change the predicted structures (Data not shown). We then analyzed the effects of the mutations on replication activity. The m3 C substitution in JFH-1 VR was found to reduce replication activity 100-fold of the J6/N3H+5BSLX-JFH1-Luc (Fig. 4D; VRm3), whereas other substitutions (Fig. 4A; m1, m2 and m4) did not reduce replication activity at all (Fig. 4D; VRm1, VRm2 and VRm4). In contrast, the construct containing the J6CF VR with m3 G substitution completely restored replication activity (Fig. 4D; VR-J6m3). Other JFH-1 type nucleotide did not restore replication activity (Fig. 4D; VR-J6m1, VR-J6m2 and VR-J6m4). These results were in agreement with the stem-loop structure prediction of VR (Fig. 4E), demonstrating that the JFH-1 VR increased RNA replication. These results suggested the importance of VR secondary structure. Next, we tested if the effect of VR of JFH-1 was restricted to NS5B of JFH-1 or not. We constructed replicons with NS5B of J6CF and tested the effect on replication. The replication activities of the replicon with entire NS5B of J6CF (J6/N3H+3′UTR-JFH1), J6/N3H+3′UTR-JFH1 with A450S or Y561F (J6/N3H+3′UTR-JFH1+A450S, J6/N3H+3′UTR-JFH1+Y561F, respectively) were enhanced by the VR of JFH-1 (see polyU-J6 of each constructs in Fig. 4C) and not enhanced by the VR of J6CF (see VR-J6 of each constructs in Fig. 4C). These results indicated that the VR structure of JFH-1 was preferable for both JFH-1- and J6CF-derived NS5B and this effect was independent of the enhanced kissing-loop interaction (compare J6/N3H+3′UTR-JFH1 wt and A450S vs. Y561F in Fig. 4C).

## Discussion

It has been demonstrated previously that HCV JFH-1, the only strain that replicates and produces virions efficiently in cell culture systems, expresses high replication activity without adaptive mutations [8]. We have previously reported that the N3H and N5BX regions of JFH-1 were able to rescue replication of the genotype 2a replicons [12]. The NS3 helicase and N5BX regions have been shown to be important to the virus production in HuH-7 cells. We have continued this line of experiment in the current study by focusing on RdRP activity and the genome structure in the 5BSL3.2 (CRE) to 3′X region. Following these aims, we were able to define the amino acids, nucleotides, and structural elements of JFH-1 required to confer replication competence and replication efficiency to the closely related J6CF.

In the present study, we identified five JFH-1-type amino acid residues in NS5B (Q377R, A450S, S455N, R517K, and Y561F) important for HCV replication by the *in vitro* polymerase activity assay and *in vivo* assays using replicons and full length HCV RNA. These amino acid residues are all in the thumb domain of HCV RdRP. All of these JFH-1-type substitutions increased the polymerase activity of J6CF RdRP. J6CF-type amino acids substitution into JFH-1 RdRP, including R377Q, S450A, K517R, and F561Y, reduced polymerase activity, while the N455S substitution demonstrated similar activity to the JFH-1 wt. The A450S and S455N substitutions resulted in the most significant enhancement of 1b [13] and J6CF RdRP (Fig. 1), respectively. aa 450 is located at the tip of the β-hairpin, while aa 455 is located close to the lower portions of the β-hairpin that may control the entry of the RNA template [13]. Both the β-hairpin (aa 450 to 455) and the β-strand (aa 560 to 565) of the thumb domain play an important role in RNA binding due to their extensive hydrogen-bonding network [23]. The β-hairpin has been shown to prevent the recruitment of the primer-template complex into the RNA-binding site to ensure accurate initiation from the 3′ end of the template [24], [25]. A450S and S455N are thought to possibly affect J6CF RdRP structure by changing the spacing of the nucleic acid binding pocket occluded by the β-hairpin. As JFH-1 N455S did not decrease the polymerase activity of JFH-1, the thumb domain of JFH-1 may be optimized to control the position or movement of the β-hairpin. Simister *et al.* have recently reported that the higher *in vitro* polymerase activity of JFH-1 was due to a higher *de novo* initiation efficiency that may be due to a closed conformation of the JFH-1 polymerase [26]. Eight amino acid mutations in NS5B of JFH-1 are hypothesized to be responsible for the conformational differences in the NS5B sequences JFH-1 and the 2a consensus [26]. However, these amino acids did not overlap with the mutations that we identified to be important for replication. Taken together, these two studies suggested that the thumb structure surrounding the β-hairpin is important to RdRP activity [26]. We only tested six of 29 amino acid differences and other mutations are possibly important to RdRP activity. However, SKF and SNKF slightly increased replication activity compared to the replicon with entire NS5B of JFH-1 (Fig. 2C). These results suggest that there may be some JFH-1-type variants in NS5B region that inhibit the replication activity of JFH-1. The JFH-1 and J6CF 5BSL regions (Fig. S2) differ in three amino acids. The JFH-1-type substitution R517K and Y561F increased replication, while the variation at aa 571 did not affect replication. This means that there are no JFH-1 variants in 5BSL region that inhibit replication activity. However, some other mutations which were not tested outside of 5BSL region may inhibit replication. Taken together, we considered that is why the replicon with 5BSLX of JFH-1 had almost the same replication activity as the replicon with entire NS5B region of JFH-1.

After comparing the activating effects of A450S and S455N vs. R517K and Y561F in the *in vitro* polymerase, *in vivo* RNA replication and virus production assays, we hypothesized that amino acids 517 and 561 likely control HCV genome replication via interactions with additional host and viral factors, including the NS3 helicase and 3′UTR. A450S enhances polymerase activity alone, while R517K and Y561F enhance genome transcription and replication activity via additional factors. The aa 455 and 517 are known to be located at the surface of the polymerase, and these mutations may affect interactions with the proteins that play important roles in RNA replication.

The combination of A450S, R517K, and Y561F substitutions conferred replication activity to the replicon with J6CF RdRP. The results of the core production were in agreement with the results from the replicon assay and suggested that these amino acid mutations affected only RNA replication and did not affect the additional steps in the virus life cycle within the cells, such as virus particle assembly and virus secretion.

We did, however, observe a discrepancy between the effects of the mutations on *in vitro* RNA polymerase activity and *in vivo* RNA replication and virus production activities. The S455N mutation conferred the highest levels of activity on J6CF RdRP *in vitro*, while Y561F conferred the highest replication and virus production activities on J6/N3H+3′UTR-JFH1 in the cells. We did not observe any combination effects of the substitutions in the *in vitro* polymerase assays, while strong combination effects of the substitutions were observed on replication and core production *in vivo*. In addition, the combination of only three substitutions (SKF; A450S, R517K, and Y561F) was enough to increase HCV replication to levels similar to that of the construct harboring both the entire NS5B region and the 3′UTR of JFH-1. We did not observe any combination effects of the substitutions in the *in vitro* polymerase assays using 1b RdRP [13]. However, a discrepancy between polymerase activity *in vitro* and replication activity was also reported for GTP binding site mutants [27].

Discrepancies between the results from *in vitro* polymerase activity assays and *in vivo* replication assays may arise because of differences in the assay systems. In an *in vitro* polymerase assay, only enzymatic activity can be determined, while an *in vivo* assay of replication activity does not necessarily represent the only polymerase activity. Many viral and host factors may be involved in the RNA replication step in the cells. If a HCV replication assay using entirely reconstituted components were possible, we could compare the isolated effect of different polymerase variants on polymerase activity.

In addition to RdRP activity, host and viral factors, including *cis*-acting RNA structures in the 3′-genome must be considered in HCV replication in cells. In fact, we found a JFH-1-type nucleotide variant in NS5B region important to maintain the genome structure in the *in vivo* assay; this *cis*-acting factor could not have been identified using the *in vitro* polymerase assay. The SKF triple substitution contains the 561F variant that is important for enhanced kissing-loop interaction and high polymerase activity, suggesting that the effects of the SKF combination *in vivo* are rather due to the enhanced kissing-loop interaction.

We also analyzed the 5BSL3.2 and 3′XSL2 structures required for kissing-loop interactions, as aa 561 is in the loop domain of 5BSL3.2 and the activation effect of Y561F in the *in vivo* replicon assay was larger than in the *in vitro* polymerase assay. In order to test the effects of JFH-1-type variants of 5BSL3.2 on replication, we substituted the amino acids located downstream of the 5BSL3-to-3′X region (nt 9211 to 9678) from JFH-1 into the J6CF construct carrying the JFH-1-type NS3 helicase (J6/N3H+5BSLX-JFH1). The J6/N3H+5BSLX-JFH1 exhibited similar replication and virus production levels to J6/N3H+N5BX-JFH1. We initially focused on the amino acid differences between JFH-1 and J6CF in the region spanning between JFH-1 5BSL-to-3′X because this region was able to complement the entire JFH-1 NS5B-to-3′ X region. We identified three amino acid differences (517, 561, and 571) in the 5BSLX regions of JFH-1 and J6CF. We then introduced J6CF-type substitutions into the 5BSL3.2 region of JFH-1 RdRP. The J6CF-type substitution in JFH-1 5BSL3.2 region at positions 517 and 561, but not 571, resulted in a reduction in replication. These findings were consistent with the results of the *in vitro* polymerase assay. RNA polymerase activity *in vitro* was analyzed using the ΔC21-molecule (1–570) and JFH-1 RdRP that did not contain 571S demonstrated high levels of polymerase activity, indicating that 571S may not be important for its high polymerase activity. The codon encoding aa 517 is located outside of the 5BSL3.2 region, suggesting that this mutation only affected polymerase activity. The codon encoding aa 561 and aa 571 are within the 5BSL3.2 region. The codon encoding aa 561 is located within the loop of the 5BSL3.2, while the codon encoding aa 571 is in the spacer region located between 5BSL3.2 and 5BSL3.3. The nucleotide mutations resulting the K517R and S571L aa substitutions were predicted to maintain 5BSL3.2 RNA secondary structures similar to that of JFH-1 using Mfold analysis [21].

Since there was the possibility that Y561F mutation affected both RdRP protein activity and genomic RNA structures, we tested the effect of nucleotide substitutions in the aa 561 codon on replication. The third nucleotide (9349U) contained within the codon encoding aa 561 is conserved among the different genotypes and essential for the kissing-loop interaction [16], [22]. The second nucleotide (nt 9348) of JFH-1 is a U, while that of J6CF an A. The first nucleotide (nt 9347) of the codon should be either an A or a U, because these nucleotides are required to maintain the loop structure. Thus, a Phe (JFH-1), Tyr (J6CF and 1b), or Ile (9347A) may reside at position 561. As JFH-1 RdRP F561I retained identical activity levels to the wt (561F), hydrophobic amino acids appeared to be required in this position to maintain the high polymerase activity. Since the predicted secondary structures of 5BSL3.2 were identical for JFH-1 and J6CF, both Phe located at position 561 and the nucleotide sequence UUU in JFH-1 were essential for the high replication activity in cultured cells.

The conserved sequences of the kissing-loop interaction were UCACAGC (nt 9349–9355) in 5BSL3.2 and GCUGUGA (nt 9612–9618) in 3′X SL2. In the case of JFH-1, the nucleotide located at position 9348 was U and the nucleotide located at position 9619 was A, resulting in extended kissing-loop interaction sequence in JFH-1. When we introduced a mutation into the 3′X SL2 region (nt 9619) that was expected to abolish the extra base pair next to the interaction site, replication activity was significantly decreased. In addition, a combination of the F561Y and 3′X SL2m substitutions, expected to restore the extra base pair between nt 9348 and nt 9619, restored replication. Replication level of this double substitution was slightly lower than that of the wt constructs, possibly due to the preference for Phe at 561 over Tyr for genome replication. Mfold analysis also revealed that RNA secondary structure was not affected following the introduction of these substitutions. U at nucleotide position 9348 was previously identified in various HCV strains registered in GenBank [7]. Taken together, these findings suggested that the strong kissing-loop interaction of the JFH-1 genome supports efficient genome replication in HuH-7 cells. We also tested the effect of Y561F substitution in two other genotypes, H77S (GT1a) and HCV-N (GT1b). While the Y561F substitution increased replication activity in both genotype 1 strains, the Y561F effect on the genotype 1 strains was much smaller than its corresponding effect on J6CF. These results may indicate that the levels of Y561F effect for viral RNA replication are different among the genotypes. These results may also indicate that the Y561F substitution enhanced replication of strains with a substantial replication capacity. In case of J6CF, the Y561F effect was only observed with N3H region and VR of JFH-1 (Fig. 4C, compare VR-J6 and polyU-J6 of J6/N3H+3′UTR-JFH1+Y561F). This result suggested that the Y561F effect was difficult to detect with replication-incompetent clones or clones with weak replication, and also suggested that other mutations or regions are important to replicate genotype 1 replicon efficiently. Therefore, we need more efficient replicating clone of genotype 1 to determine the effect and importance of this mutation on genotype 1 strains.

We next analyzed the effects of 3′UTR structure on replication and demonstrated that the polyU/UC of JFH-1 was 27 nucleotides shorter than that of J6CF. The shorter polyU/UC and the RNA structure of the VR of JFH-1 appeared enhance efficient replication. When using the activated RdRP (SKF) and the optimal RNA structure of the 3′ genome together with the JFH-1 NS3 helicase, we found that J6CF, which did not replicate in cells, was successfully converted to a replicating virus. The VR sequence is generally not conserved, even among strains within the same genotype, and the effects of VR on HCV replication remain controversial [14], [15], [28]. Our data revealed that the VR of JFH-1 was more favorable than that of J6CF for replication. Substitution of the VR from JFH-1 to J6CF significantly reduced replication levels 1000-fold. This dramatic change in replication activity was likely due to alterations in the RNA structure with a mutation at nt 9458. The predicted RNA structure of the VR and replication activity of the constructs containing substitutions or mutations to the VR were completely correlated. It is therefore very likely that cellular and viral factors interact with the HCV genome in this region, and that the specific nucleotide sequence and higher structure of the VR may be essential for these interactions. There is a possibility of genetic interaction between the VR and NS5B region. These kinds of interaction may also affect on polymerase activity.

The length of the polyU/UC tract appeared to be flexible and even differed within the same genotype. Even though JFH-1 and J6CF shared an identical 3′X, the JFH-1 poly U/UC tract (nt 9483–nt 9579) was 27 U shorter than that of J6CF (nt 9483–nt 9606). Thus, we examined whether the polyU/UC tract could be exchanged between JFH-1 and J6CF. The J6/N3H+5BSLX-JFH1 variant that contained the J6CF polyU/UC exhibited a four-fold reduction in replication, demonstrating that the polyU/UC did indeed affect replication. Several published papers have investigated the affects of length on the polyU/UC region [14], [15], [16]. Several viral and cellular proteins have also been reported to interact with the polyU sequence [29], [30], [31], [32], [33], [34], [35], [36]. The preferential length and nucleotide sequence of the polyU/UC may be determined by interaction with these factors.

In conclusion, we found that high RdRP activity, enhanced kissing-loop interaction between 5BSL3.2 and 3′X SL2, optimal VR structure and a shorter polyU/UC tract in JFH-1 contributed to the high levels of HCV RNA replication and virus production in cultured cells. As NS3 helicase region of JFH-1 is also important for replication and viral production of J6CF, the replication enhancing mechanism of NS3 helicase region should be analyzed.

## Supporting Information

Figure S1 (A). Purified HCV J6CF and JFH-1 mutant RNA polymerases. HCV RdRp variants were purified as indicated in the Materials and Methods section. Five pmol of RdRp were applied on 10% SDS-PAGE and stained with Coomassie brilliant blue. The designations of HCV J6CF and JFH-1 wt and mutants are indicated above the PAGE. M; molecular weight marker (Takara), and the position is indicated on the left. (B). Representative PAGE of *in vitro* transcription of HCV J6CF and JFH-1 mutant RNA polymerases. *In vitro de novo* transcription was performed as indicated in the Materials and Method section. [^32^P]-RNA products were applied on 6% PAGE containing 8 M urea. The autoradiography was analyzed by Typhoon trio plus image analyzer. The radio isotope count of 184 nt RNA product was measured and compared to that of JFH-1 RdRp wt in the same PAGE. The designations of HCV J6CF and JFH-1 wt and mutants are indicated above the PAGE. M; [^32^P]-25 base DNA ladder (Takara), and the position is indicated on the left. The position of 184 nt RNA product is indicated on the right.
(0.45 MB TIF)

Figure S2 Comparisons of the amino acid sequence of NS5B of JFH-1 and J6CF. The 5BSL region is indicated with a box.
(0.13 MB TIF)

Figure S3 Effect of Y561F substitution on replication activity of genotype 1 replicons. Replication activity of genotype 1a (H77S: (A)) and 1b (HCV-N:(B)) replicons. Subgenomic RNA was synthesized *in vitro* from wild-type or chimeric replicon constructs. Transcribed subgenomic RNA (5 µg) was then electroporated into HuH-7 cells and the cells serially harvested 4, 24, and 48 h after transfection. The harvested cells were lysed and the luciferase activity of the cell lysates was measured. The assays were performed three times independently, and the results expressed as luciferase activities (RLU). Luciferase activity is expressed as the change in RLU (n-fold) relative to the luciferase activity 4 h after transfection. Each value was corrected for transfection efficiency as determined by measuring the luciferase activity 4 h after transfection. Data are presented as the mean ± standard deviation for luciferase activity.
(0.08 MB TIF)

Table S1 Oligonucleotides used for construction.
(0.04 MB XLS)

Text S1 Supplementary materials and methods
(0.03 MB DOC)

## References

[1] Lemon S, Walker C, Alter M, Yi M, Knipe D, Howley P (2007). Hepatitis C virus.. Fields Virology 5 ed.

[2] Wasley A, Alter MJ (2000). Epidemiology of hepatitis C: geographic differences and temporal trends.. Semin Liver Dis.

[3] Grakoui A, Wychowski C, Lin C, Feinstone SM, Rice CM (1993). Expression and identification of hepatitis C virus polyprotein cleavage products.. J Virol.

[4] Hijikata M, Mizushima H, Tanji Y, Komoda Y, Hirowatari Y (1993). Proteolytic processing and membrane association of putative nonstructural proteins of hepatitis C virus.. Proc Natl Acad Sci U S A.

[5] Tsukiyama-Kohara K, Iizuka N, Kohara M, Nomoto A (1992). Internal ribosome entry site within hepatitis C virus RNA.. J Virol.

[6] Tanaka T, Kato N, Cho MJ, Shimotohno K (1995). A novel sequence found at the 3′ terminus of hepatitis C virus genome.. Biochem Biophys Res Commun.

[7] You S, Stump DD, Branch AD, Rice CM (2004). A cis-acting replication element in the sequence encoding the NS5B RNA-dependent RNA polymerase is required for hepatitis C virus RNA replication.. J Virol.

[8] Wakita T, Pietschmann T, Kato T, Date T, Miyamoto M (2005). Production of infectious hepatitis C virus in tissue culture from a cloned viral genome.. Nat Med.

[9] Zhong J, Gastaminza P, Cheng G, Kapadia S, Kato T (2005). Robust hepatitis C virus infection in vitro.. Proc Natl Acad Sci U S A.

[10] Lindenbach BD, Evans MJ, Syder AJ, Wolk B, Tellinghuisen TL (2005). Complete replication of hepatitis C virus in cell culture.. Science.

[11] Pietschmann T, Kaul A, Koutsoudakis G, Shavinskaya A, Kallis S (2006). Construction and characterization of infectious intragenotypic and intergenotypic hepatitis C virus chimeras.. Proc Natl Acad Sci U S A.

[12] Murayama A, Date T, Morikawa K, Akazawa D, Miyamoto M (2007). The NS3 helicase and NS5B-to-3′X regions are important for efficient hepatitis C virus strain JFH-1 replication in Huh7 cells.. J Virol.

[13] Weng L, Du J, Zhou J, Ding J, Wakita T (2009). Modification of hepatitis C virus 1b RNA polymerase to make a highly active JFH1-type polymerase by mutation of the thumb domain.. Arch Virol.

[14] Friebe P, Bartenschlager R (2002). Genetic analysis of sequences in the 3′ nontranslated region of hepatitis C virus that are important for RNA replication.. J Virol.

[15] Yanagi M, Purcell RH, Emerson SU, Bukh J (1999). Hepatitis C virus: an infectious molecular clone of a second major genotype (2a) and lack of viability of intertypic 1a and 2a chimeras.. Virology.

[16] You S, Rice CM (2008). 3′ RNA elements in hepatitis C virus replication: kissing partners and long poly(U).. J Virol.

[17] Nakabayashi H, Taketa K, Miyano K, Yamane T, Sato J (1982). Growth of human hepatoma cells lines with differentiated functions in chemically defined medium.. Cancer Res.

[18] Kuiken C, Combet C, Bukh J, Shin IT, Deleage G (2006). A comprehensive system for consistent numbering of HCV sequences, proteins and epitopes.. Hepatology.

[19] van den Hoff MJ, Moorman AF, Lamers WH (1992). Electroporation in ‘intracellular’ buffer increases cell survival.. Nucleic Acids Res.

[20] Kato T, Date T, Miyamoto M, Sugiyama M, Tanaka Y (2005). Detection of anti-hepatitis C virus effects of interferon and ribavirin by a sensitive replicon system.. J Clin Microbiol.

[21] Zuker M (2003). Mfold web server for nucleic acid folding and hybridization prediction.. Nucleic Acids Res.

[22] Friebe P, Boudet J, Simorre JP, Bartenschlager R (2005). Kissing-loop interaction in the 3′ end of the hepatitis C virus genome essential for RNA replication.. J Virol.

[23] Leveque VJ, Johnson RB, Parsons S, Ren J, Xie C (2003). Identification of a C-terminal regulatory motif in hepatitis C virus RNA-dependent RNA polymerase: structural and biochemical analysis.. J Virol.

[24] Zhong W, Ferrari E, Lesburg CA, Maag D, Ghosh SK (2000). Template/primer requirements and single nucleotide incorporation by hepatitis C virus nonstructural protein 5B polymerase.. J Virol.

[25] Hong Z, Cameron CE, Walker MP, Castro C, Yao N (2001). A novel mechanism to ensure terminal initiation by hepatitis C virus NS5B polymerase.. Virology.

[26] Simister P, Schmitt M, Geitmann M, Wicht O, Danielson UH (2009). Structural and functional analysis of hepatitis C virus strain JFH1 polymerase.. J Virol.

[27] Cai Z, Yi M, Zhang C, Luo G (2005). Mutagenesis analysis of the rGTP-specific binding site of hepatitis C virus RNA-dependent RNA polymerase.. J Virol.

[28] Arumugaswami V, Remenyi R, Kanagavel V, Sue EY, Ngoc Ho T (2008). High-resolution functional profiling of hepatitis C virus genome.. PLoS Pathog.

[29] Kanai A, Tanabe K, Kohara M (1995). Poly(U) binding activity of hepatitis C virus NS3 protein, a putative RNA helicase.. FEBS Lett.

[30] Luo G, Hamatake RK, Mathis DM, Racela J, Rigat KL (2000). De novo initiation of RNA synthesis by the RNA-dependent RNA polymerase (NS5B) of hepatitis C virus.. J Virol.

[31] Huang L, Hwang J, Sharma SD, Hargittai MR, Chen Y (2005). Hepatitis C virus nonstructural protein 5A (NS5A) is an RNA-binding protein.. J Biol Chem.

[32] Gontarek RR, Gutshall LL, Herold KM, Tsai J, Sathe GM (1999). hnRNP C and polypyrimidine tract-binding protein specifically interact with the pyrimidine-rich region within the 3′NTR of the HCV RNA genome.. Nucleic Acids Res.

[33] Luo G (1999). Cellular proteins bind to the poly(U) tract of the 3′ untranslated region of hepatitis C virus RNA genome.. Virology.

[34] Petrik J, Parker H, Alexander GJ (1999). Human hepatic glyceraldehyde-3-phosphate dehydrogenase binds to the poly(U) tract of the 3′ non-coding region of hepatitis C virus genomic RNA.. J Gen Virol.

[35] Spangberg K, Wiklund L, Schwartz S (2001). Binding of the La autoantigen to the hepatitis C virus 3′ untranslated region protects the RNA from rapid degradation in vitro.. J Gen Virol.

[36] Spangberg K, Wiklund L, Schwartz S (2000). HuR, a protein implicated in oncogene and growth factor mRNA decay, binds to the 3′ ends of hepatitis C virus RNA of both polarities.. Virology.

