# Supplementary figures and images for: RNA Polymerase Activity and Specific RNA Structure Are Required for Efficient HCV Replication in Cultured Cells
Source: PLoS Pathog. 2010 Apr 29;6(4):e1000885. doi: 10.1371/journal.ppat.1000885 (PMC2861710; doi:10.1371/journal.ppat.1000885)

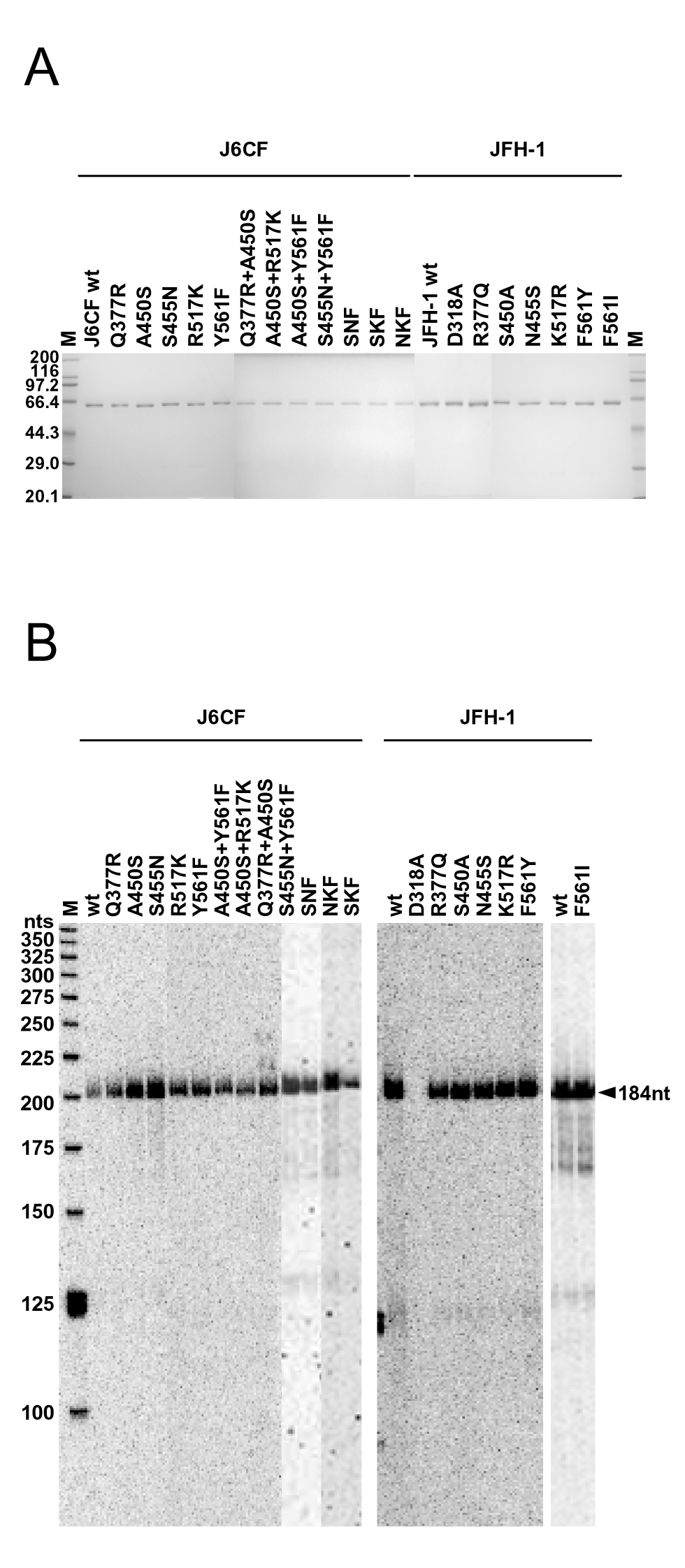

Supplement: Figure S1 — (A). Purified HCV J6CF and JFH-1 mutant RNA polymerases. HCV RdRp variants were purified as indicated in the Materials and Methods section. Five pmol of RdRp were applied on 10% SDS-PAGE and stained with Coomassie brilliant blue. The designations of HCV J6CF and JFH-1 wt and mutants are indicated above the PAGE. M; molecular weight marker (Takara), and the position is indicated on the left. (B). Representative PAGE of in vitro transcription of HCV J6CF and JFH-1 mutant RNA polymerases. In vitro de novo transcription was performed as indicated in the Materials and Method section. [32P]-RNA products were applied on 6% PAGE containing 8 M urea. The autoradiography was analyzed by Typhoon trio plus image analyzer. The radio isotope count of 184 nt RNA product was measured and compared to that of JFH-1 RdRp wt in the same PAGE. The designations of HCV J6CF and JFH-1 wt and mutants are indicated above the PAGE. M; [32P]-25 base DNA ladder (Takara), and the position is indicated on the left. The position of 184 nt RNA product is indicated on the right. (0.45 MB TIF) [file ppat.1000885.s001.tif]

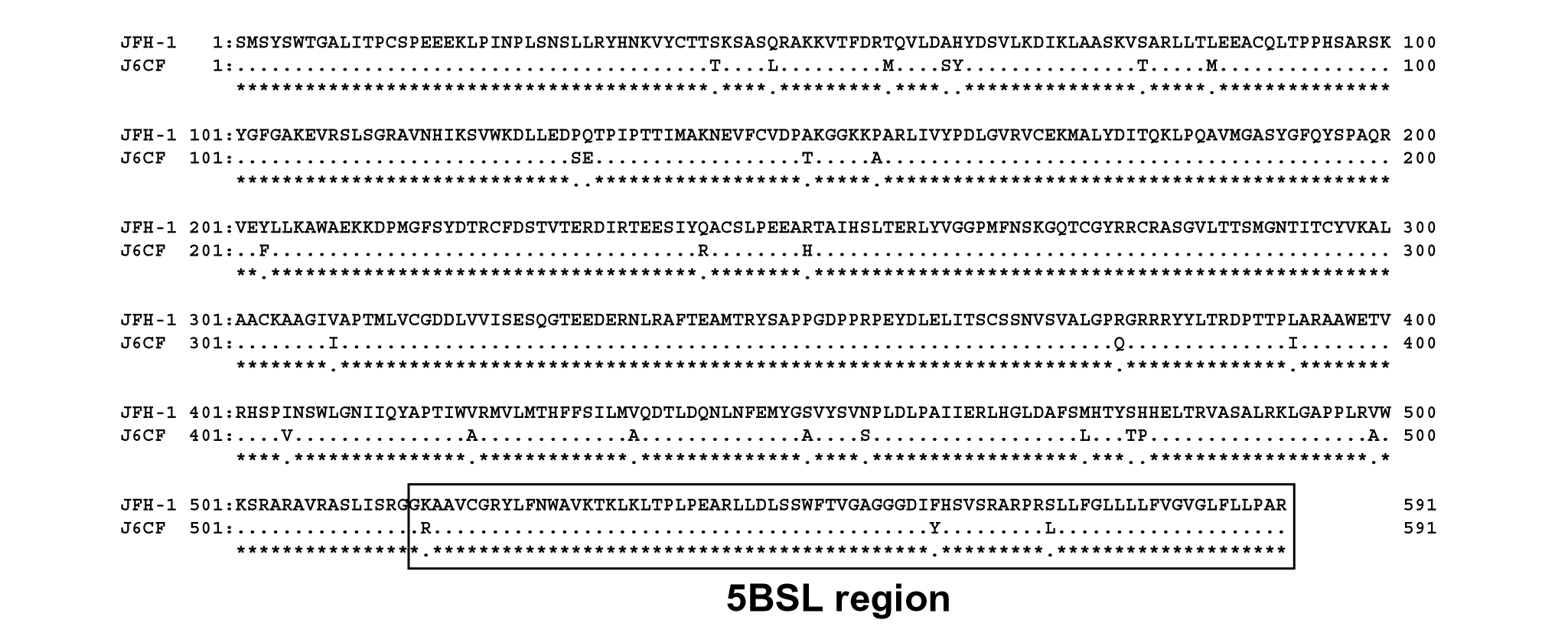

Supplement: Figure S2 — Comparisons of the amino acid sequence of NS5B of JFH-1 and J6CF. The 5BSL region is indicated with a box. (0.13 MB TIF) [file ppat.1000885.s002.tif]

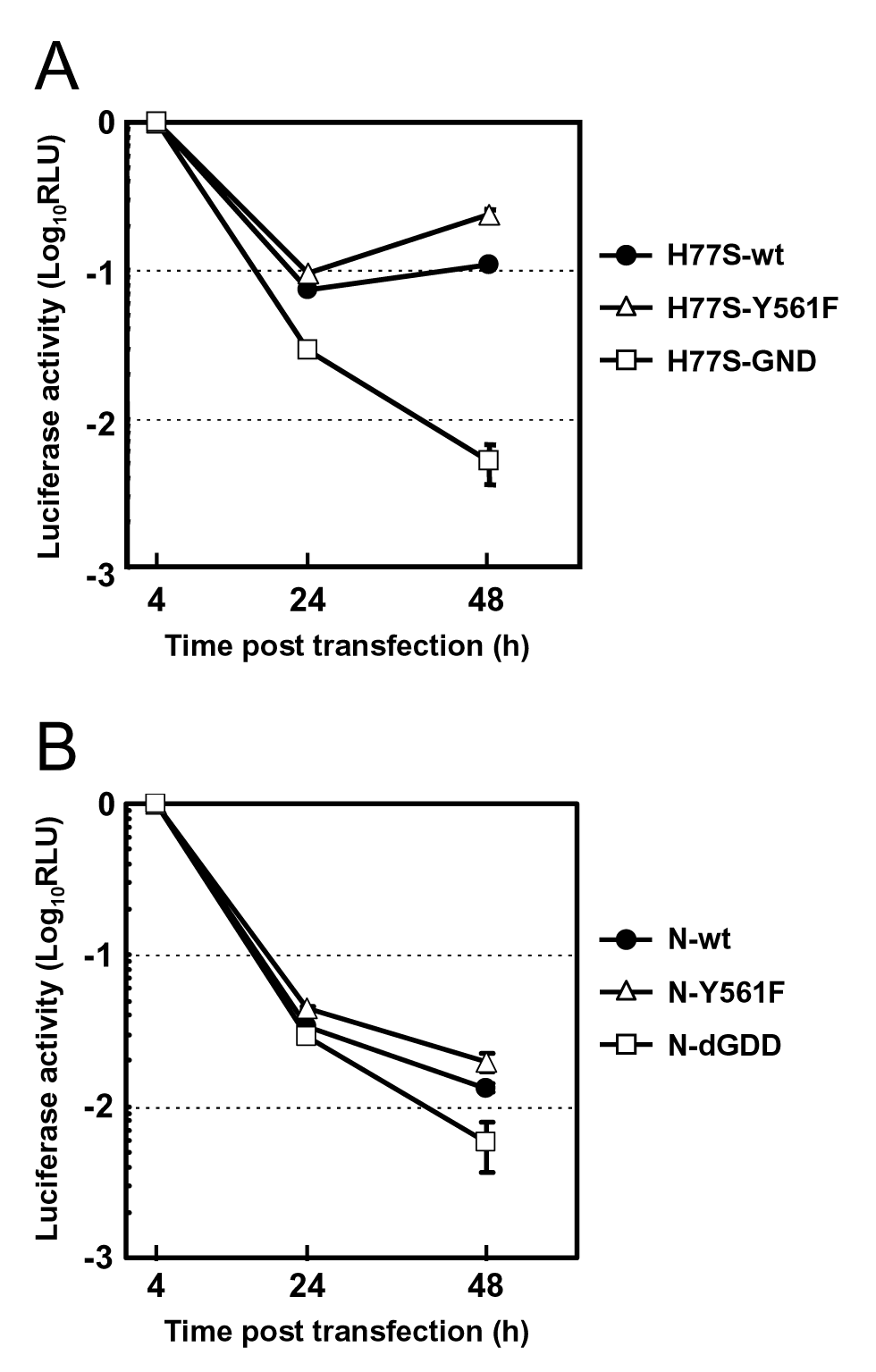

Supplement: Figure S3 — Effect of Y561F substitution on replication activity of genotype 1 replicons. Replication activity of genotype 1a (H77S: (A)) and 1b (HCV-N:(B)) replicons. Subgenomic RNA was synthesized in vitro from wild-type or chimeric replicon constructs. Transcribed subgenomic RNA (5 µg) was then electroporated into HuH-7 cells and the cells serially harvested 4, 24, and 48 h after transfection. The harvested cells were lysed and the luciferase activity of the cell lysates was measured. The assays were performed three times independently, and the results expressed as luciferase activities (RLU). Luciferase activity is expressed as the change in RLU (n-fold) relative to the luciferase activity 4 h after transfection. Each value was corrected for transfection efficiency as determined by measuring the luciferase activity 4 h after transfection. Data are presented as the mean ± standard deviation for luciferase activity. (0.08 MB TIF) [file ppat.1000885.s003.tif]
