# Supplementary material for: RNA Polymerase Activity and Specific RNA Structure Are Required for Efficient HCV Replication in Cultured Cells
Source: PLoS Pathog. 2010 Apr 29;6(4):e1000885. doi: 10.1371/journal.ppat.1000885 (PMC2861710; doi:10.1371/journal.ppat.1000885)
Supplement: Text S1 — Supplementary materials and methods (0.03 MB DOC) [file ppat.1000885.s005.doc]

**Supplementary Materials and Methods.**

**Subgenomic-replicon constructs.**

pH77-S and H77c are different at five RdRP amino acids. To generate H77S-Luc, the first mutation was introduced by PCR-based mutagenesis and the rest were introduced by exchanging *Sgr*AI-*Bgl*II fragment of H77c-Luc [1] with pH77-S [2]. H77S-GND-Luc and H77S-Y561F-Luc were constructed by PCR-based mutagenesis. To obtain HCV-N replicons bearing luciferase reporter gene (N-Luc and N-dGDD-Luc), *Hind*III-restricted fragment of pNNeo/3-5B and pNNeo/3-5BΔGDD [3] were exchanged with a corresponding fragment of pFK-I389/luc/NS3-3'/NK5.1 [4]. N-Y561F-Luc was generated by PCR-based mutagenesis.

**References of Supporting Information**

1. Murayama A, Date T, Morikawa K, Akazawa D, Miyamoto M, et al. (2007) The NS3 helicase and NS5B-to-3'X regions are important for efficient hepatitis C virus strain JFH-1 replication in Huh7 cells. J Virol 81: 8030-8040.

2. Yi M, Villanueva RA, Thomas DL, Wakita T, Lemon SM (2006) Production of infectious genotype 1a hepatitis C virus (Hutchinson strain) in cultured human hepatoma cells. Proc Natl Acad Sci U S A 103: 2310-2315.

3. Ikeda M, Yi M, Li K, Lemon SM (2002) Selectable subgenomic and genome-length dicistronic RNAs derived from an infectious molecular clone of the HCV-N strain of hepatitis C virus replicate efficiently in cultured Huh7 cells. J Virol 76: 2997-3006.

4. Lohmann V, Hoffmann S, Herian U, Penin F, Bartenschlager R (2003) Viral and cellular determinants of hepatitis C virus RNA replication in cell culture. J Virol 77: 3007-3019.
